# The Causative Pathogen Determines the Inflammatory Profile in Cerebrospinal Fluid and Outcome in Patients with Bacterial Meningitis

**DOI:** 10.1155/2013/312476

**Published:** 2013-06-20

**Authors:** Denis Grandgirard, Rahel Gäumann, Boubacar Coulibaly, Jean-Pierre Dangy, Ali Sie, Thomas Junghanss, Hans Schudel, Gerd Pluschke, Stephen L. Leib

**Affiliations:** ^1^Neuroinfection Laboratory, Institute for Infectious Diseases, University of Bern, Friedbuehlstraße 51, 3010 Bern, Switzerland; ^2^Centre de Recherche en Sante de Nouna, Nouna, Burkina Faso; ^3^Swiss Tropical and Public Health Institute and University of Basel, 4051 Basel, Switzerland; ^4^Section of Clinical Tropical Medicine, Heidelberg University Hospital, 69120 Heidelberg, Germany; ^5^Biology Division, Spiez Laboratory, Federal Office for Civil Protection (FOCP), 3700 Spiez, Switzerland

## Abstract

*Background*. The brain's inflammatory response to the infecting pathogen determines the outcome of bacterial meningitis (BM), for example, the associated mortality and the extent of brain injury. The inflammatory cascade is initiated by the presence of bacteria in the cerebrospinal fluid (CSF) activating resident immune cells and leading to the influx of blood derived leukocytes. To elucidate the pathomechanisms behind the observed difference in outcome between different pathogens, we compared the inflammatory profile in the CSF of patients with BM caused by *Streptococcus pneumonia* (*n* = 14), *Neisseria meningitidis* (*n* = 22), and *Haemophilus influenza* (*n* = 9). *Methods*. CSF inflammatory parameters, including cytokines and chemokines, MMP-9, and nitric oxide synthase activity, were assessed in a cohort of patients with BM from Burkina Faso. *Results*. Pneumococcal meningitis was associated with significantly higher CSF concentrations of IFN-**γ**, MCP-1, and the matrix-metalloproteinase (MMP-) 9. In patients with a fatal outcome, levels of TNF-**α**, IL-1**β**, IL-1RA, IL-6, and TGF-**α** were significantly higher. *Conclusion*. The signature of pro- and anti-inflammatory mediators and the intensity of inflammatory processes in CSF are determined by the bacterial pathogen causing bacterial meningitis with pneumococcal meningitis being associated with a higher case fatality rate than meningitis caused by *N. meningitidis* or *H. influenzae.*

## 1. Introduction

The three major pathogens causing bacterial meningitis (BM) are *Streptococcus pneumoniae *(*SP*), *Haemophilus influenzae type b *(*Hib*) and *Neisseria meningitidis *(*NM*). BM is the most severe and frequent infection of the central nervous system (CNS) and is associated with a high mortality rate and adverse neurological outcome in a substantial proportion of survivors [1]. BM caused by SP has the highest case fatality and neurological disability rates compared to those caused by NM or Hib [2, 3]. In a recent systematic review, the median inhospital case fatality ratio among African children with BM was 35% for SP, 25% for Hib, and 4% for NM meningitis [4]. In addition, about a quarter of children surviving pneumococcal meningitis and Hib meningitis had neuropsychological sequelae by the time of hospital discharge.

A number of factors have been identified as predictive for a poor outcome in terms of mortality. Coma and seizures were found to be predictive, next to shock, peripheral circulatory failure, severe respiratory distress, a low peripheral white blood cell (WBC) count, and a high CSF protein level in a recent systematic review of prognostic studies [5]. 

The host inflammatory reaction in the CNS is initiated by the recognition of the invading pathogens and results in the local production of soluble mediators. Differences in the innate immune responses upon stimulation with Gram-positive and Gram-negative bacteria have been demonstrated in vitro and in experimental infection models. These differences are presumably related to pathogen-specific activation of pattern recognition receptors [6–9]. Brain cells, that is, astrocytes, microglial cells, endothelial cells, ependymal cells, and resident macrophages, react to the invading pathogens by releasing early response inflammatory cytokines, like IL-1*β*, TNF-*α*, and IL-6. TNF-*α* stimulates the recruitment of neutrophils and monocytes to the sites of infection and activates these cells to eliminate pathogens, by releasing reactive molecules, amongst others NO. Antibiotics causing rapid lysis of the bacteria have been shown to exacerbate CSF inflammation by increasing TNF-*α* [10]. After stimulation by bacterial wall components or TNF-*α*, IL-1*β* is released by mononuclear phagocytes, glial cells, and endothelial cells. High CSF level of IL-1*β* significantly correlates with adverse outcome and severity of BM [11]. Administration of TNF-*α* or IL-1*β* into the CSF results in pathophysiological changes characteristic of BM [12, 13]. IL-6 is produced by monocytes, endothelial cells, and astrocytes, mainly in response to IL-1*β* [14]. IL-10 and IL-1RA antagonize the effect of proinflammatory cytokines or chemokines, by inhibiting their production (IL-10) or acting as a decoy receptor (IL-1RA). CSF levels of other cytokines and chemokines (IL-2, IL-8, IFN-*γ*, MCP-1, MIP-1, and G-CSF) have also been found elevated in BM [15–19]. White blood cells invading the CSF release MMPs and reactive molecules [20–22] which are critically involved in the pathogenesis of brain damage in BM. Therapeutic strategies targeting MMPs and oxidative radical have yielded promising results, albeit limited to experimental BM models [20–24] to date. Tissue-destructive agents released by leukocytes and brain resident cells, like matrix-metalloproteinases (MMPs) and oxidants, also mediate brain damage in BM [22]. In BM, MMPs are involved in the blood-brain barrier opening, in immune cell extravasation, in the release of cytokines and cytokine receptors, and in the development of neuronal damage. In patients, elevated CSF levels of MMP-9 and MMP-8 have been detected [21], and high levels of MMP-9 were identified as a risk factor for sequelae [25]. Nitric oxide (NO) has been shown to contribute to the pathophysiology of meningitis with a phase-dependent role at the level of the cerebral vasculature by hyperemic effects in early phase and vasodilative effects protecting against ischemia in later phase [26]. 

Since all of the above-detailed inflammatory mediators have been shown to influence the outcome in experimental models of BM, we set out to determine in patients with BM the association between the CSF concentration of these mediators with the causative organism and the mortality. To this end the pathogen-specific inflammatory profiles caused by *S. pneumoniae*, *N. meningitidis*, and *H. influenzae* were analyzed in CSF from BM patients. The findings from this study may help understand, at a pathophysiological level, the difference in outcome observed between the different pathogens.

## 2. Materials and Methods

### 2.1. CSF Samples

CSF samples were collected in the Nouna Health District (NHD), Burkina Faso, during two consecutive meningitis seasons [27]. Ethical clearance for the meningitis study was obtained from the “Comite Local d'Ethique de Nouna” (Nouna Local Ethical Committee). Procedures followed were in accordance with the ethical standards of the committee and with the Helsinki Declaration of the World Medical Association. Informed consent was obtained from all study participants. Following the national guidelines for meningitis surveillance, diagnostic lumbar puncture was performed on patients with a suspicion of meningitis presenting to one of the 25 health centers of the NHD. Patients were enrolled into the study if their CSF could be transported on ice and analyzed by trained personnel in the laboratory of Nouna District Hospital within 6 hours. For primary analysis, white blood cell counts were determined. Samples were tested for bacterial pathogens using Gram staining, culture, latex agglutination, or PCR. CSF samples were centrifuged to remove white blood cells and supernatants stored at −80°C. Samples with conflicting diagnostics for the etiological agent between culture and PCR were excluded from the analysis. During transport from Africa to Switzerland, samples were kept frozen in liquid nitrogen.

CSF samples with confirmed acute BM (positive culture and/or positive PCR, CSF WBC of more than 50∗10^6^ cells/liter) were categorized into three analytical groups according to the causative agent: *S. pneumoniae* (SP, *n* = 14), *N. meningitidis* (NM, *n* = 22), and *H. influenzae type b* (Hib, *n* = 9).

### 2.2. Assessment of Cytokine Levels in CSF Samples

Cytokine levels in CSF samples were assessed using microsphere-based multiplex assays (Lincoplex, LINCO Research Inc., St. Charles, MA, USA). CSF concentrations of the following cytokines were measured: IL-1*α*, IL-1*β*, IL-2, IL-6, IL-8, IL-10, IL-1RA, IFN-*γ*, MCP-1, MIP-1*α*, MIP-1*β*, TGF-*α*, and TNF-*α*. To fit the dynamic range of the test, samples were assessed undiluted or diluted 5- to 25-fold with the provided assay buffer, depending on the expected concentration of the respective analytes to be tested, as determined in preliminary experiments. A minimum of 50 beads per analyte was measured. Calibration curves from the provided standards were calculated using BioPlex Manager software version 4.1.1 with a five-parametric logistic curve fitting. When measured cytokine concentrations were below the detection limit, a value corresponding to the detection limit of the assay multiplied by the dilution factor of the sample was used for statistical analysis. 

Validation of the assay was done for IL-10 and TNF-*α* using Enzyme-linked immunosorbent assays (ELISA) (R&D Systems Inc., Minneapolis, MN, USA). According to the concentrations estimated using the Lincoplex assay and the sensitivity range of the ELISA, samples were diluted 10-fold (TNF-*α*) or 20-fold (IL-10) to a final volume of 200 *μ*L using the appropriate calibrator diluent. Results obtained by Luminex and conventional ELISA were compared for correlation, using Prism Software. For both cytokines, a significant correlation was found between the two methods (TNF-*α*, *P* = 0.002, Spearman *r* = 0.53; IL-10, *P* < 0.0001, Spearman *r* = 0.75).

### 2.3. Assessment of MMP-9 Levels in CSF Samples

MMP-9 levels were assessed using the Fluorokine MAP Human MMP Kit (R&D Systems Inc., Minneapolis, MN, USA). All CSF samples were diluted 100-fold, to a final volume of 50 *μ*L. A minimum of 50 beads was measured. Standard curves were calculated similarly to those of the cytokines assay. 

Validation of the assay was done using gelatin-containing gel zymography as already described [21]. Concentrations measured by the Fluorokine MAP assay correlated with those assessed by gelatin zymography with a two-tailed *P*-value of <0.0001 (Spearman's rank correlation test, *r* = 0.66).

### 2.4. Measurement of Total Nitrate and Nitrite in CSF Samples

CSF levels of total nitrate and nitrite were assessed using a nitrate/nitrite colorimetric assay (Cayman Chemical Company, Ann Arbor, MI, USA). The estimated concentrations were used as an index for nitric oxide synthase activity. CSF samples were filtered for 30 min at 10000 g using Ultrafree −0.5 centrifugal filter devices. Samples and assay buffer (each 40 *μ*L) were mixed with 10 *μ*L of coenzyme mixture and 10 *μ*L of nitrate reductase in a 96-well plate. After 3 h at room temperature (RT) for conversion of nitrate to nitrite, Griess reagents were added for 10 min at RT. Absorbance was measured at 550 nm. Total nitrite concentrations were calculated using standard curves generated by the SoftMax PRO software version 3.1.2 (Molecular Devices Inc., Sunnyvale, CA, USA) using a linear curve fitting.

### 2.5. Statistical Analysis

Statistical analysis was done using GraphPad Prism version 5.04 (GraphPad Software Inc., La Jolla, CA, USA). For comparison of the different pathogen groups, we first tested whether data sets followed a Gaussian distribution. At least one group did not follow a Gaussian distribution for each comparison. Furthermore, since we had to include arbitrary values, the nonparametric Kruskal-Wallis test was used. If the overall test was significant (*P* < 0.05), the Mann-Whitney test was applied to perform pairwise comparisons. For the analysis of the relation between CSF cytokine levels and the outcome of the disease, the nonparametric Mann-Whitney test was used, with a confidence interval of 95% and two-tailed *P* values. Correlations were analyzed using Spearman's rank correlation test, with a confidence interval of 95% and two-tailed *P* values.

## 3. Results

### 3.1. Clinical Parameters

Significant pathogen-specific differences in the age distribution of patients were observed within the study cohort (*P* Kruskal-Wallis test: *P* < 0.01). NM meningitis was found in patients 0–60 years (median: 5.5 years, *n* = 22) and SP meningitis in patients 0–40 years (median: 5.5 years, *n* = 14). In contrast, Hib meningitis affected only children 1–4 years (median: 2 years, *n* = 9). The difference in median ages was significant between SP versus Hib (*P* < 0.05) and NM versus Hib (*P* < 0.01). Mortality of BM patients was 46% for SP and 27% for NM, while all nine patients infected with Hib survived (Table 1).

CSF white blood cell counts did not significantly differ within the 3 groups (median SP: 7020 × 10^6^/L [100–64000]; median NM: 4900 × 10^6^/L [100–38560]; median Hib: 5540 × 10^6^/L [272–20000]) (Table 2). 

### 3.2. Cytokine and Chemokine Levels in CSF

**Figure 1:**
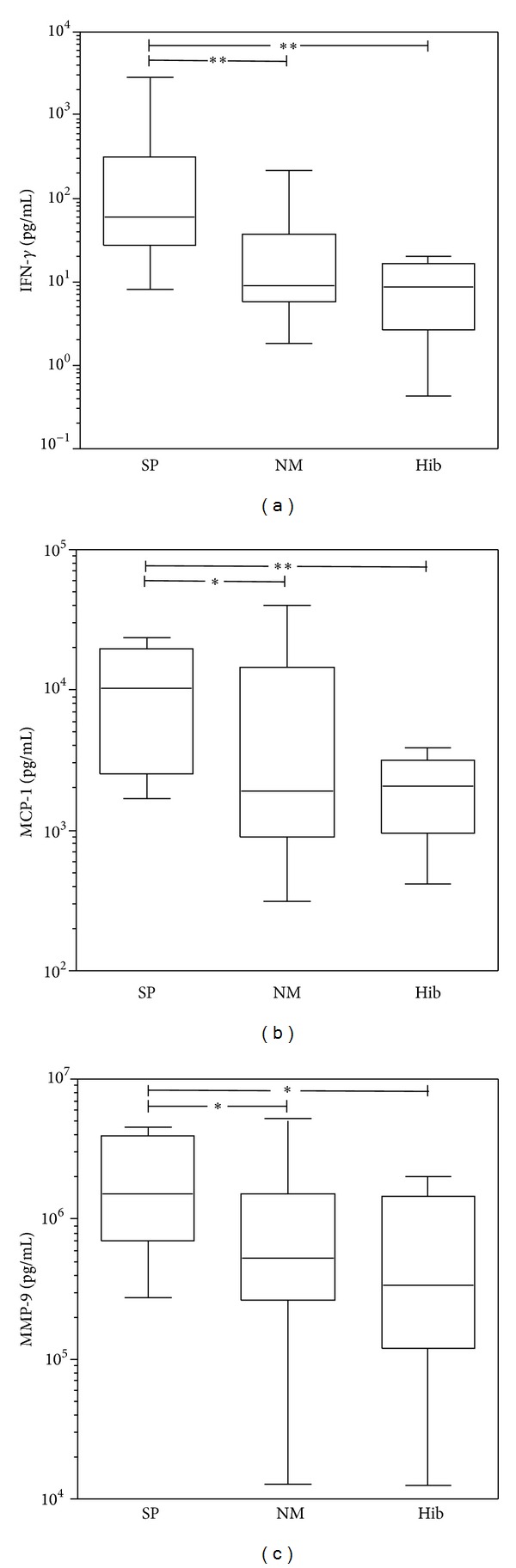
Inflammatory CSF parameters in BM patients. Statistically significant differences (*P < 0.05; **P < 0.01) in CSF concentrations of MCP-1, IFN-*γ*, and MMP-9 were observed in patients with BM grouped for the causative pathogens.

**Figure 2:**
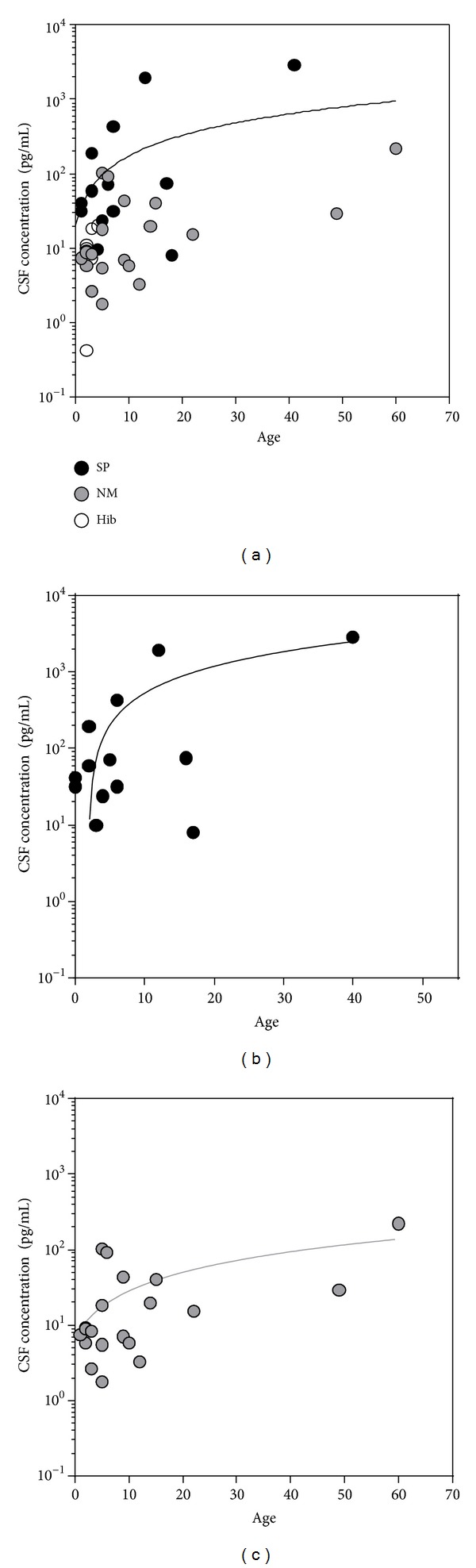
Correlation between CSF levels of IFN-*γ* and age of the patients. (a) The level of IFN-*γ* correlated with the age of the patients. This correlation was significant for SP patients ((b): *P* = 0.01, *r* = 0.79, and black dots) and NM patients ((c): *P* = 0.02, *r* = 0.64, and grey dots).

**Figure 3:**
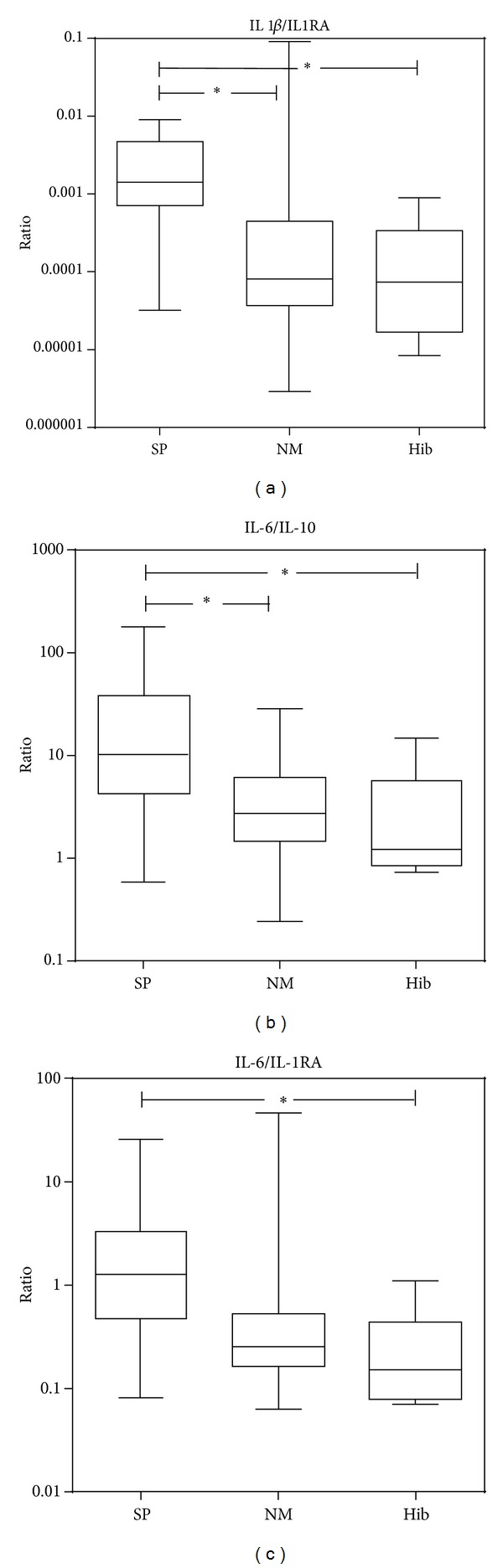
Ratio of pro- to anti-inflammatory mediators. Statistically significant differences (*P < 0.05) in the ratios of IL-1*β*/IL-1RA, IL-6/IL-10, and IL-6/IL-1RA were observed in patients with BM grouped for the causative pathogens.

Cytokines and chemokines showed significantly different regulations between the causative bacteria (Table 2 and Figure 1). In particular, the CSF concentration of IFN-*γ* was significantly higher in patients infected with SP compared to NM (*P* < 0.005) and Hib (*P* < 0.005). The CSF concentration of IFN-*γ* correlated with the age of the patients (*P* = 0.15, Pearson's *r* = 0.38; Figure 2). This correlation was significant for meningitis caused by SP (*P* = 0.01, *r* = 0.79) and NM (*P* = 0.02, *r* = 0.64). This suggests that, during meningitis, adults are more apt to react with IFN-*γ* production than children [28]. Since there was a statistically significant difference in age for the Hib group, we cannot exclude that the difference in IFN-*γ* level in this population may be due to the age, rather than the pathogen. For MCP-1 significant differences between SP versus NM patients (*P* = 0.045) and SP versus Hib patients (*P* < 0.01) were observed (Table 2). In addition, a nonsignificant trend for SP causing higher levels of IL-1*β* (*P* < 0.07) and IL-6 (*P* = 0.055) was found. Taken together, reciprocal trends in the association of pro- and anti-inflammatory cytokines and chemokines with BM caused by the different pathogens were observed. Il-1*β*, IFN-*γ*, and MCP-1, as prototypical proinflammatory factors, showed higher CSF concentrations in the patients infected with SP than by NM and Hib. In contrast, the anti-inflammatory mediators IL-10 and IL-1RA, were more increased in CSF of patients infected with NM and Hib. The ratio of pro- to anti-inflammatory mediators, in particular the IL-1*β*/IL-1RA ratio, showed statistically significant differences, being higher in SP versus NM (*P* < 0.01) and SP versus Hib (*P* < 0.03). Similar correlations were found for IL-6/IL-10 and IL-6/IL-1RA ratios (Figure 3). Cyto-/chemokines concentrations were significantly higher in patients infected with any of the 3 pathogens when compared with a group of 7 healthy control patients, as defined by no clinical signs of meningitis and no increase in WBC in the CSF (median 4 × 10^6^ cells/L). For IL-1*β*, IL-2, TNF*α*, IFN*γ*, and MIP1 *α*, the majority of these samples were under detection limit, even when samples were analyzed undiluted (Table 3).

**Figure 4:**
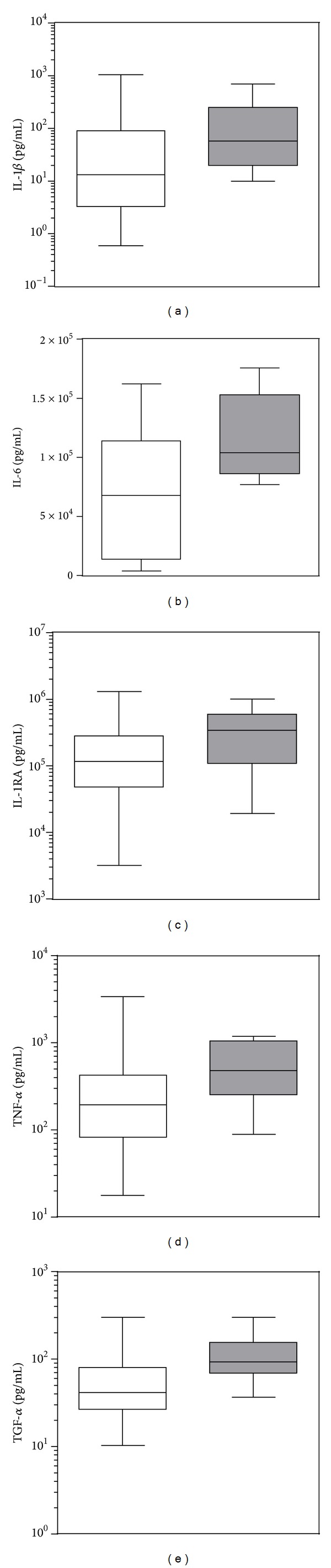
Significant differences in cytokines CSF levels in relation to disease outcome. Patients were grouped independently of the etiological agents based on the outcome (nonlethal, white boxes/lethal, grey boxes) of the disease. Pairwise comparisons (Mann-Whitney test) revealed statistically significant differences for the 5 cytokines (see also Table 4).

Since the host inflammatory reaction during BM is an important determinant of disease severity and mortality, the association between CSF cytokine and chemokine levels and outcome (survival or death) was investigated (Table 4(a) and Figure 4). When all patients were analyzed together, a significant association between fatal outcome and CSF levels of 5 cytokines, namely, IL-1*β*, TNF-*α*, IL-1RA, IL-6 (*P* < 0.05), and TGF-*α* (*P* < 0.02), was found. When pathogens were investigated separately, TNF-*α* was significantly higher in patients who died from SP meningitis (Table 4(b)), while only IL-1RA was significantly higher in patients with a fatal outcome after NM meningitis (Table 4(c)). 

### 3.3. MMP-9, Nitrate and Nitrite Levels in CSF

In children with bacterial meningitis, matrix-metalloproteinase- (MMP-) 9 in the cerebrospinal fluid has been associated with blood-brain barrier damage and neurological sequelae [25]. Concentrations of MMP-9 were highest in CSF of patients suffering from SP meningitis (*P* < 0.03; Table 1 and Figure 1). Pairwise comparisons between the different etiological agents revealed SP versus Hib to differ significantly (*P* < 0.02) as well as SP versus NM (*P* < 0.04) (Figure 1). MMP-9 showed a nonsignificant trend towards higher CSF levels in patients who died from the disease (Mann-Whitney test: *P* = 0.068, Table 4(a)). 

Levels of total nitrate and nitrite showed a nonsignificant trend (*P* ≤ 0.06) between pathogens, with higher CSF concentrations in samples of NM patients than in SP and Hib patients (Table 1).

## 4. Discussion

In addition to the high mortality of up to 30%, cases of BM and specifically those caused by SP are associated with persistent neurological sequelae in up to 50% of the survivors due to different forms of brain damage [29, 30]. The burden of disease is especially high in low-income countries, and risk of mortality or major sequelae is twice as high in African as in the European regions [31]. Over the last four decades, the risk of major postdischarge sequelae caused by meningitis has not significantly changed [31]. Both clinical and experimental studies suggest that both the pathogen and the inflammatory host response contribute to the development of mortality and neurological sequelae.

Here we compared the host immune response in the CSF to BM caused by *S. pneumoniae*, *N. meningitidis*, and *H. influenzae*. To date, only few studies have compared the CSF concentration of inflammatory mediators during BM in relation to the bacterial pathogen [17, 28, 32]. Here we found that the pathogen is an important determinant of the inflammatory CSF reaction to BM. The observed difference in inflammation in the CSF may not only be due to inherent differences between pathogens to elicit a response in cells of the innate immune system [6–9] but also due to the ability of the pathogen to multiply in the CNS compartment. Unfortunately, determining the bacterial load in the CSF of patients was not feasible in the present study. 

In accordance with published data [17, 28], we observed significantly higher CSF concentration of IFN-*γ* in pneumococcal meningitis. Furthermore, as observed by others, the level of IFN-*γ* correlated with the age of the patients. This suggests that, during meningitis, adults are more apt to react with IFN-*γ* production than children [28]. IFN-*γ* is a potent proinflammatory cytokine. It enhances the function of macrophages and polymorphonuclear leukocytes by stimulating nonspecific defense mechanisms such as phagocytosis and the release of inflammatory mediators and may therefore contribute to the overshooting inflammation. 

We found elevated levels of the chemokines MCP-1, MIP-1*α*, and MIP-1*β* in the CSF of patients with BM, in accordance with other published studies [18, 19, 33]. CSF concentrations of MCP-1 were significantly higher in patients infected with SP compared to both NM and Hib. Elevated CSF levels of MCP-1 have been shown to exacerbate brain damage during neuroinflammatory diseases by increasing the influx of monocytes and neutrophils [34]. However, we did not find a correlation of MCP-1 levels with WBCs in the CSF, which is in accordance with other studies [18, 33]. Furthermore, WBC count did not correlate with any other parameters, either when pathogen groups were pooled together or when analyzed separately. Worsening of the outcome by an overshooting inflammatory reaction is further suggested by the increased ratios of pro- to anti-inflammatory mediators (IL-6/IL-10, IL6/IL-1RA, and IL-1*β*/IL-1RA) observed in patients infected by SP. In a previous study in BM patients, CSF showed higher ratio of TNF-*α*/IL-10 by SP when compared to NM and Hib combined [17].

High CSF concentration of MMP-9 is a risk factor for a detrimental outcome [25, 35, 36]. Our results add further support to the notion that MMP-9 is critically involved in the increase in mortality and sequelae, since CSF levels of MMP-9 were significantly higher in BM caused by SP, which is usually associated with a higher incidence of neurological sequelae and mortality [25]. 

When the relationship between inflammatory mediators and the outcome of the disease was investigated independently of the causative agent, higher CSF levels of TNF-*α*, IL-1*β*, IL-1RA, IL-6, and TGF-*α* were found in patients who died from BM. A nonsignificant trend was also found for MMP-9 and IL-6. Thus, the present study confirms previous observations showing that both a strong activation of the IL-1*β* system [37] and increased MMP-9 levels [25] correlate with adverse outcome of BM. While the higher mortality observed in SP may be seen in the context of higher cytokine levels, this observation could not be made for the difference in mortality between NM (27%) and Hib (0%) where inflammatory CSF parameters were not significantly different. In comparison to other studies in which mortality in developing regions reached 30% [38], the mortality attributed to Hib was exceptionally low in the present study. The small group size for Hib is a clear limitation of the study. Furthermore, NM meningitis is more often associated with fulminant septicemia, which may contribute to mortality. Unfortunately, data concerning the presence of concomitant septicemia were not available in the present study. Interestingly, the inhibition of the metalloproteinase TACE/ADAM17, acting as a sheddase for TNF-*α* and TGF-*α* [39], has been shown to lower mortality and to attenuate brain injury in experimental models of BM [20, 36]. In line with these results, we could show in the present study that TNF-*α* was significantly upregulated in patients with a poor outcome. In accordance with this clinical observation is the experimental finding that deletion of another member of the TGF family, TGF-*β* has been shown to improve bacterial clearance and diminished intracranial complication in a mouse with pneumococcal meningitis [40].

A nonsignificant trend for higher levels of nitrite/nitrate levels was observed in NM infected patients. In experimental models, CSF NO/nitrite concentration correlated with an increase in blood-brain barrier permeability, but inhibition of the different nitric oxide synthases resulted in inconsistent effects, probably as a result of differences in the timing of intervention and the corresponding effects on the brain perfusion. 

The present study, analyzing a cohort of patients affected by meningitis, identified several factors which contribute to the worsening of outcome in bacterial meningitis. Interestingly, some of these factors (TNF-*α*, MMPs, and nitric oxide) have already been described in experimental models using knockout animals [41] and/or intervention strategies [42] which reduced mortality and ameliorated the outcome of infected animals. The most promising strategies derived from these experimental models include the reduction of the inflammatory reaction by targeting different steps in the inflammatory process [43], from the release of proinflammatory bacterial products to the activation of the innate immune system and the production/release of cytokines or chemokines, as well as the inhibition of metalloproteinases or treatments with antioxidants [44]. 

## 5. Conclusion

In conclusion, this study showed that SP, NM, and Hib elicit distinct profiles of inflammatory mediators in the CSF during BM. A more intense inflammatory reaction, in particular higher CSF levels of IFN-*γ*, MCP-1, and MMP-9, were observed in patients infected with SP. Furthermore, the ratios of pro- to anti-inflammatory parameters were found to be significantly higher in patients with SP meningitis. This is likely to contribute to the higher case fatality rate and morbidity observed in patients suffering from pneumococcal meningitis and may therefore help find new treatment strategies aimed at improving the outcome of infected patients. 

## Figures and Tables

**Table 1:** Patient groups characteristics.

Pathogen	Number of patients	Gender (male/female)	Median age (min–max)	Mortality (%) (survivors/death)
*S. pneumoniae *	14	5/8^1^	5.5 (0–40)	46% (7/6)^1^
*N. meningitidis *	22	10/9^2^	5.5 (1–60)	27% (16/6)
*H. influenzae *	9	7/2	2 (1–4)	0% (9/0)

^1^1 patient not documented.

^2^3 patients not documented.

**Table 2:** CSF inflammatory parameters of patients infected with SP (*n* = 14), NM (*n* = 22), and Hib (*n* = 9).

	*Streptococcus pneumoniae *	*Neisseria meningitidis *	*Haemophilus influenzae *	*P* Kruskal-Wallis	Between group significance
	Median (min.–max.) (pg/mL)	Median (min.–max.) (pg/mL)	Median (min.–max.) (pg/mL)		
IL-1*β*	116.2 (1.79–1040)	23.01 (0.61–325.9)	13.3 (0.77–112.5)	0.069	(b)
IL-2	3.28 (0.38–19.81)	1.41 (0.38–25.69)	1.6 (0.18–3.83)	ns	
IL-6	106232 (4440–175553)	93316 (5917–162002)	23257 (3917–112594)	0.0551	(b)
IL-10	7515 (509.5–44609)	18604 (1883–217931)	12692 (1466–135980)	ns	
IL-1RA	103123 (4413–983243)	243089 (3183–639929)	216530 (7712–1301000)	ns	
TNF-*α*	233.8 (34.85–1199)	318.2 (17.6–3390)	170.3 (43.63–1395)	ns	
IFN-*γ*	58.99 (8.03–2853)	8.94 (1.77–219.8)	8.62 (0.42–20.03)	<0.01	(a), (b)
MCP-1	10109 (1691–23567)	1896 (309.8–40005)	2059 (411.4–3897)	<0.04	(a), (b)
MIP-1*α*	829.1 (30.75–3828)	1469 (133.8–27027)	1152 (135.6–12782)	ns	
MIP-1*β*	3344 (575.8–7518)	3263 (530.7–90852)	2563 (1364–22695)	ns	
TGF-*α*	73.4 (16.36–302.3)	65.26 (10.3–146.5)	43.23 (27.52–305.2)	ns	
MMP-9	1.51 × 10^6^ (275632–4.5 × 10^6^)	525821 (1877–5.32 × 10^6^)	334058 (12440–2.006 × 10^6^)	<0.03	(a), (b)
WBC	7020 (100–64000)	4900 (100–38560)	5540 (272–20000)	ns	
Nitrite/nitrate*	19.8 (9.5–104)	37 (19.24–124.1)	21.36 (4.533–41.5)	0.059	(c)

The nitrite/nitrate concentration (NO) was determined in a subset of CSFs, due to limitations in the available sample volumes (Hib *n* = 8, SP = 8, and NM = 12). The column entitled “Between group significance” describes statistical significance between groups as determined by Mann-Whitney test, for the following comparisons (a) SP versus NM, (b) SP versus Hib, and (c) NM versus Hib.

**Table 3:** CSF inflammatory parameters in control patients (*n* = 7).

Analytes	Samples under detection limit	Median (pg/mL) [min.–max.]	Dilution factor	Detection limit
IL-1*β*	5/7	n.d	1 : 1	0.19
IL-2	7/7	n.d.	1 : 1	0.38
IL-6	0/7	115 [95.06–175.1]	1 : 1	0.79
IL-10	1/7	39.96 [0.41–291.3]	1 : 1	0.41
IL-1RA	0/7	184.6 [99.82–2958]	1 : 1	10.76
TNF-*α*	4/7	n.d	1 : 1	0.22
IFN-*γ*	7/7	n.d	1 : 1	0.55
MCP-1	0/7	326 [116–11355]	1 : 5	0.63
MIP-1*α*	2/7	35.43 [1.23–536.6]	1 : 1	1.23
MIP-1*β*	4/7	n.d	1 : 1	27.65
TGF-*α*	0/7	24.16 [13.77–30.3]	1 : 1	0.69
MMP-9	0/7	106.8 [10.93–1153]	1 : 10	n.d.

A median value was calculated only when the majority of samples were above the limit of detection. Control samples were measured undiluted or diluted 1 : 5, respectively, 1 : 10.

^1^Detection limit as provided by the manufacturer.

**Table:** (a) Pooled pathogens

	Nonfatal cases	Fatal cases	Mann-Whitney
	Median (min.–max.) (pg/mL)	Median (min.–max.) (pg/mL)	
*IL-1*β**	*13.19 (0.61–1040) *	*57.19 (9.84–682) *	*0.046 *
IL-2	1.6 (0.18–255.69)	1.41 (0.38–6.9)	0.6618
*IL-6 *	*67789 (3917–162002) *	*104035 (77100–175553) *	*0.0365 *
IL-10	15764 (509.5–217931)	14131 (809–90755)	0.9351s
*IL-1RA *	*117918 (3183–1.301 × *10^6^)	*337610 (19362–983243) *	*0.0209 *
*TNF-*α**	*195.1 (17.6–3390) *	*484.3 (88.41–1199) *	*0.0436 *
IFN-*γ*	11.16 (0.42–1920)	49.13 (1.77–2853)	0.1823
MCP-1	2101 (309.8–40005)	2903 (1187–20728)	0.2193
MIP-1*α*	935.6 (133.8–27027)	1166 (30.75–16122)	0.9351
MIP-1*β*	2728 (530.7–24661)	3555 (1263–90852)	0.4177
*TGF-*α**	*41.05 (10.30–305.2) *	*93.3 (36.74–302.3) *	*0.0099 *
MMP-9	463489 (1877–5.319 × 10^6^)	1.258 × 10^6^ (313541–4.497 × 10^6^)	0.0671
WBC	4600 (100–186000)	11100 (1000–42400)	0.062
Nitrite/nitrate*	137.5 (23.15–625)	226.9 (83.6–368.5)	0.3627

**Table:** (b) SP only

	Non-fatal cases (*n* = 7)	Fatal cases (*n* = 5)	Mann-Whitney
	Median (min.–max.) (pg/mL)	Median (min.–max.) (pg/mL)	
IL-1*β*	54.44 (1.79–1040)	206.9 (9.84–682)	0.5253
IL-2	7.87 (0.38–19.81)	1.6 (0.38–5.55)	0.1452
IL-6	106232 (4440–150456)	136215 (77100–175553)	0.3434
IL-10	7515 (509.5–44609)	7497 (809–17392)	0.6010
IL-1RA	55240 (4413–275000)	121576 (19362–983243)	0.1591
*TNF-*α**	*70.65 (0.22–570.9) *	*416.2 (88.41–1199) *	*0.048 *
IFN-*γ*	40.82 (8.03–1920)	190.7 (23.73–2853)	0.202
MCP-1	10109 (1691–23059)	2897 (2020–20307)	0.8207
MIP-1*α*	829.1 (256.9–3828)	416.4 (30.75–1738)	0.5025
MIP-1*β*	2732 (575.8–6685)	3771 (1263–7342)	0.6313
TGF-*α*	35.52 (16.36–153.5)	100.5 (36.74–302.3)	0.149
MMP-9	1.103 × 10^6^ (275632–4.254 × 10^6^)	2.676 × 10^6^ (475112–4.497 × 10^6^)	0.2331
WBC	4600 (100–64000)	12930 (3200–42400)	0.1375

**Table:** (c) NM only

	Non-fatal cases (*n* = 15)	Fatal cases (*n* = 5)	Mann-Whitney
	Median (min.–max.) (pg/mL)	Median (min.–max.) (pg/mL)	
IL-1*β*	10.56 (0.61–325.9)	50.5 (10.48–205.4)	0.3056
IL-2	1.41 (0.38–25.69)	1.22 (0.38–6.9)	0.916
IL-6	67789 (5917–162002)	1100160 (77742–145357)	0.3056
IL-10	16715 (1883–217931)	47476 (5436–90755)	0.1974
*IL-1RA *	*182057 (3183–305690) *	*447933 (275000–639929) *	*0.001 *
TNF-*α*	267.2 (17.6–3390)	552.4 (247.4–1126)	0.2661
IFN-*γ*	9.1 (2.62–219.8)	8.3 (1.77–102.7)	0.5413
MCP-1	1283 (309.8–40005)	2909 (1187–20728)	0.3056
MIP-1*α*	1200 (133.8–27027)	1937 (585.1–16122)	0.444
MIP-1*β*	3137 (530.7–24661)	3388 (1851–90852)	0.5528
TGF-*α*	44.05 (10.3–146.5)	86.05 (58.93–144.7)	0.0526
MMP-9	585934 (1877–5.319 × 10^6^)	525821 (313541–2.188 × 10^6^)	0.7996
WBC	4900 (100–17000)	7800 (1000–38560)	0.3983

Significantly higher CSF concentrations in patients with a fatal outcome are represented in italic lines.

## References

[1] Grimwood K, Anderson VA, Bond L (1995). Adverse outcomes of bacterial meningitis in school-age survivors. *Pediatrics*.

[2] van de Beek D, de Gans J, Spanjaard L, Weisfelt M, Reitsma JB, Vermeulen M (2004). Clinical features and prognostic factors in adults with bacterial meningitis. *The New England Journal of Medicine*.

[3] Schuchat A, Robinson K, Wenger JD (1997). Bacterial meningitis in the United States in 1995. Active Surveillance Team. *The New England Journal of Medicine*.

[4] Ramakrishnan M, Ulland AJ, Steinhardt LC, Moïsi JC, Were F, Levine OS (2009). Sequelae due to bacterial meningitis among African children: a systematic literature review. *BMC Medicine*.

[5] de Jonge RCJ, van Furth AM, Wassenaar M, Gemke RJBJ, Terwee CB (2010). Predicting sequelae and death after bacterial meningitis in childhood: a systematic review of prognostic studies. *BMC Infectious Diseases*.

[6] Diab A, Zhu J, Lindquist L, Wretlind B, Bakhiet M, Link H (1997). Haemophilus influenzae and Streptococcus pneumoniae induce different intracerebral mRNA cytokine patterns during the course of experimental bacterial meningitis. *Clinical and Experimental Immunology*.

[7] Fowler MI, Weller RO, Heckels JE, Christodoulides M (2004). Different meningitis-causing bacteria induce distinct inflammatory responses on interaction with cells of the human meninges. *Cellular Microbiology*.

[8] Mogensen TH, Paludan SR, Kilian M, Østergaard L (2006). Live *Streptococcus pneumoniae, Haemophilus influenzae*, and **Neisseria meningitidis** activate the inflammatory response through Toll-like receptors 2, 4, and 9 in species-specific patterns. *Journal of Leukocyte Biology*.

[9] Tietze K, Dalpke A, Morath S, Mutters R, Heeg K, Nonnenmacher C (2006). Differences in innate immune responses upon stimulation with gram-positive and gram-negative bacteria. *Journal of Periodontal Research*.

[10] Mustafa MM, Ramillo O, Saez-Llorens X, Olsen KD, Magness RR, McCracken GH (1990). Cerebrospinal fluid prostaglandins, interleukin 1*β*, and tumor necrosis factor in bacterial meningitis. Clinical and laboratory correlations in placebo-treated and dexamethasone-treated patients. *American Journal of Diseases of Children*.

[11] Mustafa MM, Lebel MH, Ramilo O (1989). Correlation of interleukin-1*β* and cachectin concentrations in cerebrospinal fluid and outcome from bacterial meningitis. *Journal of Pediatrics*.

[12] Ramilo O, Saez-Llorens X, Mertsola J (1990). Tumor necrosis factor *α*/cachectin and interleukin 1*β* initiate meningeal inflammation. *Journal of Experimental Medicine*.

[13] Rosenberg GA, Estrada EY, Dencoff JE, Stetler-Stevenson WG (1995). Tumor necrosis factor-*α*-induced gelatinase B causes delayed opening of the blood-brain barrier: an expanded therapeutic window. *Brain Research*.

[14] Rusconi F, Parizzi F, Garlaschi L (1991). Interleukin 6 activity in infants and children with bacterial meningitis. *Pediatric Infectious Disease Journal*.

[15] Hackett SJ, Thomson APJ, Hart CA (2001). Cytokines, chemokines and other effector molecules involved in meningococcal disease. *Journal of Medical Microbiology*.

[16] Kastenbauer S, Angele B, Sporer B, Pfister H, Koedel U (2005). Patterns of protein expression in infectious meningitis: a cerebrospinal fluid protein array analysis. *Journal of Neuroimmunology*.

[17] Kornelisse RF, Hack CE, Savelkoul HFJ (1997). Intrathecal production of interleukin-12 and gamma interferon in patients with bacterial meningitis. *Infection and Immunity*.

[18] Mastroianni CM, Lancella L, Mengoni F (1998). Chemokine profiles in the cerebrospinal fluid (CSF) during the course of pyogenic and tuberculous meningitis. *Clinical and Experimental Immunology*.

[19] Sprenger H, Rösler A, Tonn P, Braune HJ, Huffmann G, Gemsa D (1996). Chemokines in the cerebrospinal fluid of patients with meningitis. *Clinical Immunology and Immunopathology*.

[20] Leib SL, Clements JM, Lindberg RLP (2001). Inhibition of matrix metalloproteinases and tumour necrosis factor *α* converting enzyme as adjuvant therapy in pneumococcal meningitis. *Brain*.

[21] Leib SL, Leppert D, Clements J, Täuber MG (2000). Matrix metalloproteinases contribute to brain damage in experimental pneumococcal meningitis. *Infection and Immunity*.

[22] Meli DN, Christen S, Leib SL (2003). Matrix metalloproteinase-9 in pneumococcal meningitis: activation via an oxidative pathway. *Journal of Infectious Diseases*.

[23] Auer M, Pfister L, Leppert D, Täuber MG, Leib SL (2000). Effects of clinically used antioxidants in experimental pneumococcal meningitis. *Journal of Infectious Diseases*.

[24] Leib SL, Kim YS, Chow LL, Sheldon RA, Täuber MG (1996). Reactive oxygen intermediates contribute to necrotic and apoptotic neuronal injury in an infant rat model of bacterial meningitis due to group B streptococci. *The Journal of Clinical Investigation*.

[25] Leppert D, Leib SL, Grygar C, Miller KM, Schaad UB, Holländer GA (2000). Matrix metalloproteinase (MMP)-8 and MMP-9 in cerebrospinal fluid during bacterial meningitis: association with blood-brain barrier damage and neurological sequelae. *Clinical Infectious Diseases*.

[26] Leib SL, Km YS, Black SM, Tureen JH, Täuber MG (1998). Inducible nitric oxide synthase and the effect of aminoguanidine in experimental neonatal meningitis. *Journal of Infectious Diseases*.

[27] Sié A, Pflüger V, Coulibaly B (2008). ST2859 serogroup a meningococcal meningitis outbreak in Nouna Health District, Burkina Faso: a prospective study. *Tropical Medicine and International Health*.

[28] Glimaker M, Olcen P, Andersson B (1994). Interferon-*γ* in cerebrospinal fluid from patients with viral and bacterial meningitis. *Scandinavian Journal of Infectious Diseases*.

[29] O’Brien KL, Wolfson LJ, Watt JP (2009). Burden of disease caused by *Streptococcus pneumoniae* in children younger than 5 years: global estimates. *The Lancet*.

[30] Weisfelt M, de Gans J, van der Poll T, van de Beek D (2006). Pneumococcal meningitis in adults: new approaches to management and prevention. *The Lancet Neurology*.

[31] Edmond K, Clark A, Korczak VS, Sanderson C, Griffiths UK, Rudan I (2010). Global and regional risk of disabling sequelae from bacterial meningitis: a systematic review and meta-analysis. *The Lancet Infectious Diseases*.

[32] Glimaker M, Kragsbjerg P, Forsgren M, Olcen P (1993). Tumor necrosis factor-*α* (TNF*α*) in cerebrospinal fluid from patients with meningitis of different etiologies: high levels of TNF*α* indicate bacterial meningitis. *Journal of Infectious Diseases*.

[33] Spanaus K, Nadal D, Pfister H (1997). C-X-C and C-C chemokines are expressed in the cerebrospinal fluid in bacterial meningitis and mediate chemotactic activity on peripheral blood-derived polymorphonuclear and mononuclear cells in vitro. *Journal of Immunology*.

[34] Conductier G, Blondeau N, Guyon A, Nahon J, Rovère C (2010). The role of monocyte chemoattractant protein MCP1/CCL2 in neuroinflammatory diseases. *Journal of Neuroimmunology*.

[35] Sellner J, Leib SL (2006). In bacterial meningitis cortical brain damage is associated with changes in parenchymal MMP-9/TIMP-1 ratio and increased collagen type IV degradation. *Neurobiology of Disease*.

[36] Meli DN, Loeffler JM, Baumann P (2004). In pneumococcal meningitis a novel water-soluble inhibitor of matrix metalloproteinases and TNF-*α* converting enzyme attenuates seizures and injury of the cerebral cortex. *Journal of Neuroimmunology*.

[37] van Furth AM, Roord JJ, van Furth R (1996). Roles of proinflammatory and anti-inflammatory cytokines in pathophysiology of bacterial meningitis and effect of adjunctive therapy. *Infection and Immunity*.

[38] Peltola H (2000). Worldwide Haemophilus influenzae type b disease at the beginning of the 21st century: global analysis of the disease burden 25 years after the use of the polysaccharide vaccine and a decade after the advent of conjugates. *Clinical Microbiology Reviews*.

[39] Goddard DR, Bunning RAD, Woodroofe MN (2001). Astrocyte and endothelial cell expression of ADAM 17 (TACE) in adult human CNS. *Glia*.

[40] Malipiero U, Koedel U, Pfister W, Fontana A (2007). Bacterial meningitis: the role of transforming growth factor-beta in innate immunity and secondary brain damage. *Neurodegenerative Diseases*.

[41] Paul R, Koedel U, Pfister H (2005). Development of adjunctive therapies for bacterial meningitis and lessons from knockout mice. *Neurocritical Care*.

[42] Woehrl B, Klein M, Grandgirard D, Koedel U, Leib S (2011). Bacterial meningitis: current therapy and possible future treatment options. *Expert Review of Anti-Infective Therapy*.

[43] van der Flier M, Geelen SPM, Kimpen JLL, Hoepelman IM, Tuomanen EI (2003). Reprogramming the host response in bacterial meningitis: how best to improve outcome?. *Clinical Microbiology Reviews*.

[44] Grandgirard D, Leib SL (2010). Meningitis in neonates: bench to bedside. *Clinics in Perinatology*.

